# The relationship between pomegranate root collar rot and the diversity of fungal communities in its rhizosphere

**DOI:** 10.3389/fmicb.2025.1573724

**Published:** 2025-03-21

**Authors:** Ziqiang Wu, Jianxin Chen, Jie Chen, Yalin Yang, Aiting Zhou, Jianrong Wu

**Affiliations:** ^1^Key Laboratory of Forest Disaster Warning and Control of Yunnan Province, College of Forestry, Southwest Forestry University, Kunming, China; ^2^Key Laboratory of State Forestry Administration on Biodiversity Conservation in Southwest China, College of Forestry, Southwest Forestry University, Kunming, China

**Keywords:** pomegranate, root collar rot, fungal in the pomegranate rhizosphere, the diversity and structural composition, the soil physicochemical properties

## Abstract

**Introduction:**

The pomegranate (*Punica granatum*) is a significant economic tree species. In recent years, the root collar rot has severely affected pomegranates in the dry-hot valley regions of Yunnan Province, China. The rhizosphere microbiome plays a crucial role in plant growth, development, and disease resistance.

**Methods:**

This study utilized Illumina MiSeq sequencing to analyze the fungal communities in the roots and rhizosphere soils of healthy and diseased pomegranates, focusing on the impact of root collar rot disease on the diversity and structural composition of these communities.

**Results:**

The results indicated that in the unique fungal communities of healthy plant roots, the relative abundance of ectomycorrhizal and arbuscular mycorrhizal functional (AMF) groups was 53.77%, including genera such as *Glomus* and *Septoglomus*. After infection with root collar rot disease, the rhizosphere fungal communities became more monotonous, with increased differentiation within sample groups. Fungal groups associated with plant diseases and soil nutrient structures underwent significant changes. The disease altered the composition and functional group proportions of rhizosphere fungal communities, a process linked to soil nutrient structures. And the balance between plant-pathogen-related and saprotrophic functional groups in the rhizosphere was disrupted. Through Koch’s postulates verification, the pathogen was identified as *Lauriomyces bellulus*.

**Discussion:**

This is the first report of collar rot of pomegranate caused by *L. bellulus* in China. Studying the differences in rhizosphere fungal community structures and quantities in response to new diseases aids in the rapid prediction of pathogens, effectively saving diagnostic time, and provides theoretical support for disease prediction, diagnosis, and control.

## 1 Introduction

Pomegranate (*Punica granatum* L.) has been widely cultivated for its nutritional and medicinal value, strong environmental adaptability, ease of management, and high economic benefits. In recent years, root collar rot has become increasingly prevalent in the dry-hot valley regions of Yunnan Province, China, causing significant economic losses for local fruit growers. This soilborne disease occurs when the base of the plant stem and the root collar are infected by pathogens. Internationally, Phytophthora species have been identified as common pathogens (Basım and Basım, 2013; Kurbetli et al., 2020; Ghaderi and Habibi, 2021). However, in China, there is currently no documentation of these pathogens or the associated changes in fungal community structure in the rhizosphere under disease-inducing conditions.

The rhizosphere encompasses the plant roots and surrounding soil. It is a highly dynamic region where material and information exchange occur between plants, soil, and microorganisms. Rhizosphere microorganisms are crucial for plant growth and development, and they are often the primary source of pathogens for soilborne diseases like root collar rot (Sasse et al., 2018). For instance, Li et al. (2021) utilized amplicon sequencing to compare fungal community structures in healthy and root rot infected Astragalus roots, identifying *Fusarium oxysporum* as the probable root rot pathogen, which was later confirmed through pathogenicity assays. Long-term monoculture and excessive fertilizer use disrupt soil physicochemical properties, leading to soil eutrophication and changes in microbial communities. This reduces rhizosphere fungal diversity and richness, creating conditions conducive to outbreaks of harmful microorganisms (Zuppinger-Dingley et al., 2014; Liu et al., 2015; Wang et al., 2022). Pathogen invasion or environmental changes can alter plant physiological and metabolic pathways, thereby modifying rhizodeposit composition. This, in turn, attracts specific soil microorganisms to the rhizosphere, either recruiting or reducing beneficial or pathogenic microbes, ultimately influencing plant growth and disease resistance (Berendsen et al., 2018; Yuan et al., 2018; Qi et al., 2019; You et al., 2024). For example, Berendsen et al. (2018) found that under stimulation from *Hyaloperonospora arabidopsidis*, the downy mildew pathogen, *Arabidopsis* selectively enriched three rhizosphere-specific bacteria that collaboratively induced systemic resistance against *H*. *arabidopsidis* and promoted plant growth. The abundance of key species is one of the factors reflecting microbial community functionality. In amplicon sequencing studies, dominant taxa with relative abundances greater than 1% often constitute a minority within the community but perform vital ecological roles. Rare taxa, with relative abundances below 0.1%, serve as a seed bank and contribute to nutrient cycling (Kurm et al., 2017), redox reactions (Hausmann et al., 2016), plant growth promotion, and resistance induction (Zhang et al., 2019).

This study analyzes the fungal diversity and functionality in the roots and rhizosphere soils of healthy and root collar rot-infected pomegranates. By comparing changes in fungal community structures and diversity, we identify differential species and preliminarily explore their potential ecological roles. These findings provide a foundation for identifying pathogens, understanding pathogenic mechanisms, and screening biocontrol strains for managing root collar rot in pomegranates.

## 2 Materials and methods

### 2.1 Overview of the study area and sample collection

Over the past 4 years, investigations on pomegranate root collar rot have been conducted in the dry-hot valley regions of Yunnan Province, China, and samples were collected in September 2023. Following preliminary investigations across multiple pomegranate-producing areas, a representative orchard (26°43′ N, 103°14′ E) with a certain scale was selected as the sampling site. Sampling sites were selected based on the following criteria: Consistent soil type; Representative dry-hot valley climate; Perennial pomegranate cultivation history; Uniform fertilization practices (type, rate, frequency); Root collar rot prevalence (>70% incidence); Randomized sampling design accounting for management variations.

Nine healthy pomegranate with uniform growth and nine pomegranate with consistent severity of root collar rot were selected. Roots and rhizosphere soil were collected from each plant. Samples from three plants were mixed to create one composite sample as one replicate, resulting in three composite samples per group. Healthy root samples were labeled as J1, J2, and J3, with corresponding rhizosphere soil samples labeled as K1, K2, and K3. Diseased root samples were labeled as B1, B2, and B3, with corresponding rhizosphere soil samples labeled as M1, M2, and M3. The sample types were categorized as follows: HR (healthy root), DR (diseased root), HS (healthy rhizosphere soil), and DS (diseased rhizosphere soil). The sampling method for individual plants as follow: Litter and surface soil layers were removed, and roots were excavated in a circle around the plant trunk at 20–50 cm from the trunk and 10–20 cm below the surface. Roots were extracted, and large soil clumps (over 15 cm in diameter) attached to the roots were broken apart. Fine roots and their adhering soil were collected into resealable bags and thoroughly mixed. Roots were shaken free and separated, mixed uniformly, and sealed in separate bags. Both soil and root samples were transported back to the laboratory in dry ice containers. Samples were categorized and labeled by location and replicate, then weighed, subsampled, and stored at −80°C for further analysis. Root surface sterilization was performed sequentially with: (1) 75% ethanol (40-s immersion); (2) 2.5% sodium cyclamate +0.01% Tween 80 (10-min immersion); Followed by 2–3 sterile water rinses.

### 2.2 DNA extraction and Illumina MiSeq sequencing

Total DNA was extracted from root and soil samples using the E.Z.N.A.™ Mag-Bind Soil DNA Kit (OMEGA) following the manufacturer’s protocol. The integrity of the extracted DNA was assessed using 1% agarose gel electrophoresis, and genomic DNA was accurately quantified using the Qubit 3.0 DNA Assay Kit before proceeding to PCR amplification. The primers used for PCR included sequencing platform-compatible ITS1 (5′-CTTGGTCATTTAGAGGAAGTAA-3′) and ITS2 (5′-GCTGCGTTCTTCATCGATGC-3′) (White et al., 1990). First-round PCR: The amplification was conducted in a 30 μL reaction system containing: 15 μL of 2× Hieff® Robust PCR Master Mix, 1 μL of Bar-PCR primer F, 1 μL of primer R, 10–20 ng of DNA template, ddH₂O to make up to 30 μL. The thermal cycling conditions were as follows: 94°C for 3 min (initial denaturation); 5 cycles of 94°C for 30 s, 45°C for 20 s, and 65°C for 30 s; 20 cycles of 94°C for 20 s, 55°C for 20 s, and 72°C for 30 s; Final extension at 72°C for 5 min, and storage at 10°C. Second-round PCR: To incorporate Illumina bridge PCR-compatible primers, a second round of amplification was performed in a 30 μL reaction system containing: 15 μL of 2× Hieff® Robust PCR Master Mix, 1 μL of primer F, 1 μL of Index-PCR primer R, 20–30 ng of first-round PCR product, ddH₂O to make up to 30 μL. The thermal cycling conditions were as follows: 95°C for 3 min (initial denaturation); 5 cycles of 94°C for 20 s, 55°C for 20 s, and 72°C for 30 s; Final extension at 72°C for 5 min, and storage at 10°C.

For arbuscular mycorrhizal functional (AMF), the polymerase chain reaction was performed in a 50 μL mixture as follow: 16.2 μL of 2× Hieff® Robust PCR Master Mix, 2.5 μL of each primer (AMV4.5NF 5’-AAGCTCGTAGTTGAATTTCG-3′ and AMDGR 5’-CCCAACTATCCCTATTAATCAT-3′), 4 μL of DNA template. The following thermal profile was used: 95°C for 10 min; 94°C for 30 s, 55°C for 30s, 72°C for 1 min for 35 cycles; fnally 74°C for 9 min (Sato et al., 2005).

The PCR products were analyzed for library size using 2% agarose gel electrophoresis and quantified using a Qubit 3.0 fluorometer (Hu et al., 2017). Sequencing was performed by Bioengineering (Shanghai) Co., Ltd. on the Illumina MiSeq™/HiSeq™ Illumina MiSeq sequencing platform.

### 2.3 Soil physicochemical properties analysis

The physicochemical properties of soil were determined following the methods described in the Agricultural Industry Standards of the People’s Republic of China, the Forestry Industry Standards of the People’s Republic of China.

- Soil pH: Measured using the potentiometric method.
- Soil Organic Matter (OM): Determined by the potassium dichromate dilution method.
- Available Phosphorus (AP): Measured using the bicarbonate extraction-molybdenum-antimony anti-colorimetric method.
- Available and Slow-Release Potassium (AK): Determined by ammonium acetate extraction-flame photometry.
- Total Nitrogen (TN): Measured using an automatic nitrogen analyzer.
- Total Phosphorus (TP): Determined by the sodium hydroxide fusion-molybdenum-antimony anti-colorimetric method.
- Total Potassium (TK): Measured using the hydrofluoric acid digestion method.
- Nitrate Nitrogen (NO₃^−^-N) and Ammonium Nitrogen (NH₄^+^-N): Measured using the phenol disulfonic acid colorimetric method and indophenol blue colorimetric method, respectively.

Each group consisted of three replicates, with three tests conducted for each sample. Differences among groups were assessed using one-way analysis of variance (One-Way ANOVA) to determine statistical significance.

### 2.4 Data processing

The DNA samples were sequenced on the Illumina MiSeq™/HiSeq™ platform. The raw sequencing data were processed using tools such as Cutadapt 1.18, PEAR 0.9.8, and PRINSEQ 0.20.4 for base recognition, sequence assembly, quality control, and filtering. The processed data were uploaded to the NCBI database (accession number: PRJNA1170523).

All sequences were clustered into operational taxonomic units (OTUs) using Usearch 11.0.667 at varying similarity levels. Subsequent analyses focused on OTUs clustered at a 97% similarity threshold for in-depth bioinformatic and statistical evaluations (Nguyen et al., 2016). Taxonomic annotation of OTU representative sequences was performed using the RDP Classifier Bayesian algorithm (version 2.12) and SINTAX, with database selection based on amplification types (Zhou et al., 2020). For ITS regions, UNITE was used for Blast-based annotation to identify closely related species. Community composition at phylum, class, order, family, and genus levels was summarized, and tables of relative abundance were created for fungal taxa across these taxonomic levels (Edgar, 2013; Bolger et al., 2014).

*α*-diversity indices were calculated by randomly subsampling a fixed number of sequences from each sample. Rarefaction curves were generated using R (4.2.1) to assess whether sequencing depth was sufficient, based on curve saturation. OTU clustering results were used to calculate sample library coverage and *α*-diversity indices such as the Simpson and Shannon indices using Mothur software. The *t*-test was employed to assess the significance of differences in diversity indices between groups.

Beta diversity was analyzed based on weighted normalized UniFrac distances. Principal coordinate analysis (PCoA) was conducted using the vegan package in R to visualize differences in fungal community composition between samples. Parametric tests were used to identify taxa with significant abundance differences across groups, and intergroup comparisons were performed using the Wilcoxon rank-sum test. Linear discriminant analysis (LDA) was applied to reduce data dimensionality and assess the influence of significantly different taxa, with an LDA threshold of 3. The all-against-all strategy was employed for LEfSe analysis. Redundancy analysis (RDA) was used to evaluate the relationships between environmental factors and microbial community distribution.

Analyses were performed in SPSS 26.0.0.0, and the differences among samples were assessed using a one-way ANOVA analysis, and the calculated means were subjected to Duncan’s multiple range test at *p* < 0.05.

### 2.5 Pathogenicity tests and identification of pathogen

#### 2.5.1 Preparation of conidium suspension

Plants showing aerial symptoms were categorized by disease severity. Bark tissue from root collar lesions was excised, with samples collected from both advanced infection zones (with sporulating structures) and disease fronts (symptomatic tissue without sporulation). Samples were separately bagged, labeled, and transported to the laboratory for isolation.

Symptoms on the collars of diseased pomegranate were meticulously examined using Zeiss Discovery-V20, Leica M166FC, and Leica DM2500 microscopes. Conidiophore clusters from the affected tissue were collected into sterile water. The suspension in the centrifuge tubes was gently agitated to ensure an even distribution of conidia.

#### 2.5.2 Pathogenicity tests

Pathogenicity assays were conducted on four pomegranate seedlings. The collars of these seedlings were sequentially washed with 75% ethanol, 1% sodium hypochlorite solution, and sterile water. Five small incisions were made on the young stems using a sterile needle at each of the five designated inoculation sites per seedling. Each site was then inoculated with the prepared conidium suspension. Three seedlings were treated with the suspension, while the fourth served as a control, receiving only sterile distilled water. The inoculated areas were covered with sterile, moistened absorbent cotton and sealed with a preservative film.

#### 2.5.3 Molecular characteristics of the pathogen

Genomic DNA was extracted from freshly harvested conidiophore clusters on pomegranate tissues using the CTAB method. Three ribosomal DNA regions were amplified with specific primers: NS1 and NS4 for SSU, LROR and LR7 for LSU, and ITS1 and ITS2 for ITS regions (White et al., 1990; Bunyard et al., 1994; Somrithipol et al., 2017). The resulting amplicons were sequenced by TsingKe Biotechnology. Sequence alignments and comparisons were performed using the NCBI database. Phylogenetic relationships were elucidated through Maximum Parsimony (MP) and Maximum Likelihood (ML) analyses.

## 3 Results and analysis

### 3.1 Gene sequence analysis

#### 3.1.1 Sequencing characteristics and diversity analysis

A total of 12 samples, including both healthy and diseased pomegranate roots and their corresponding rhizosphere soils, were sequenced. The Sequencing information and *α*-diversity index at genus level of fungi and AMF were showed in Table 1.

**Table 1:** Sequencing information and *α*-diversity index at genus level of root and rhizosphere soil of heathy and diseased pomegranate.

Samples/ Fungi	Raw sequences	Valid sequences	MeanLen	Simpson	Shannon	Coverage
HR	63,847 ± 5,861	63,845 ± 5,863	238.83 ± 7.99	0.11 ± 0.02b	2.91 ± 0.32b	99.8974%
DR	68,127 ± 12,952	56,563 ± 12,952	234.07 ± 10.06	0.16 ± 0.11ab	2.73 ± 0.67b	99.9389%
HS	71,649 ± 12,261	82,123 ± 12,277	239.87 ± 5.60	0.05 ± 0.01a	4.36 ± 0.16a	99.7005%
DS	62,052 ± 1,417	65,695 ± 1,413	245.64 ± 1.72	0.03 ± 0.01a	4.57 ± 0.26a	99.6495%
Total	797,027	796,920	—	—	—	—

Samples/AMF	Raw sequences	Valid sequences	MeanLen	Simpson	Shannon	Coverage
HR	57,965 ± 4,983	57,629 ± 4,865	217.16 ± 0.99	0.24 ± 0.03ab	1.99 ± 0.18ab	99.9699%
DR	57,262 ± 1,146	57,132 ± 1,269	217.18 ± 0.73	0.37 ± 0.22b	1.68 ± 0.64b	99.9389%
HS	62,317 ± 4,329	61,436 ± 4,244	221.15 ± 1.57	0.10 ± 0.01a	2.85 ± 0.09a	99.8164%
DS	55,442 ± 4,784	54,702 ± 4,878	219.53 ± 0.55	0.13 ± 0.07a	2.73 ± 0.39a	99.7451%
Total	698,962	692,699	—	—	—	—

The data in the table were means ± SD (standard deviation). Different letters in the same column indicated significant differences among samples at *p* < 0.05 level. HR: Healthy Root (healthy roots). DR, Diseased Root (roots of diseased plants); HS, Healthy Rhizosphere Soil (rhizosphere soil of healthy plants); DS, Diseased Rhizosphere Soil (rhizosphere soil of diseased plants).

At the genus level, no significant α-diversity differences was observed between diseased and healthy roots. For fungal communities, the Simpson index for healthy root samples (HR) was significantly different from that of soil samples (*p* < 0.05). In contrast, Shannon indices at the genus level were significantly higher in soil samples compared to root samples (*p* < 0.05). The similar situation was appeared of AMF communities. Fungal abundance and community diversity were greater in soil samples than in root samples, with more evenly distributed species in soil. Pomegranate roots demonstrated strong selectivity for fungal colonization. The occurrence of root collar rot did not significantly affect fungal diversity or total fungal abundance in roots and rhizosphere soils. However, based on the Simpson index, species richness in diseased root and soil samples was lower, and community composition tended to become more homogeneous. The proportion of non-dominant species decreased, while the relative abundance of potential dominant species—likely pathogens of root collar rot—increased.

**Figure 1:**
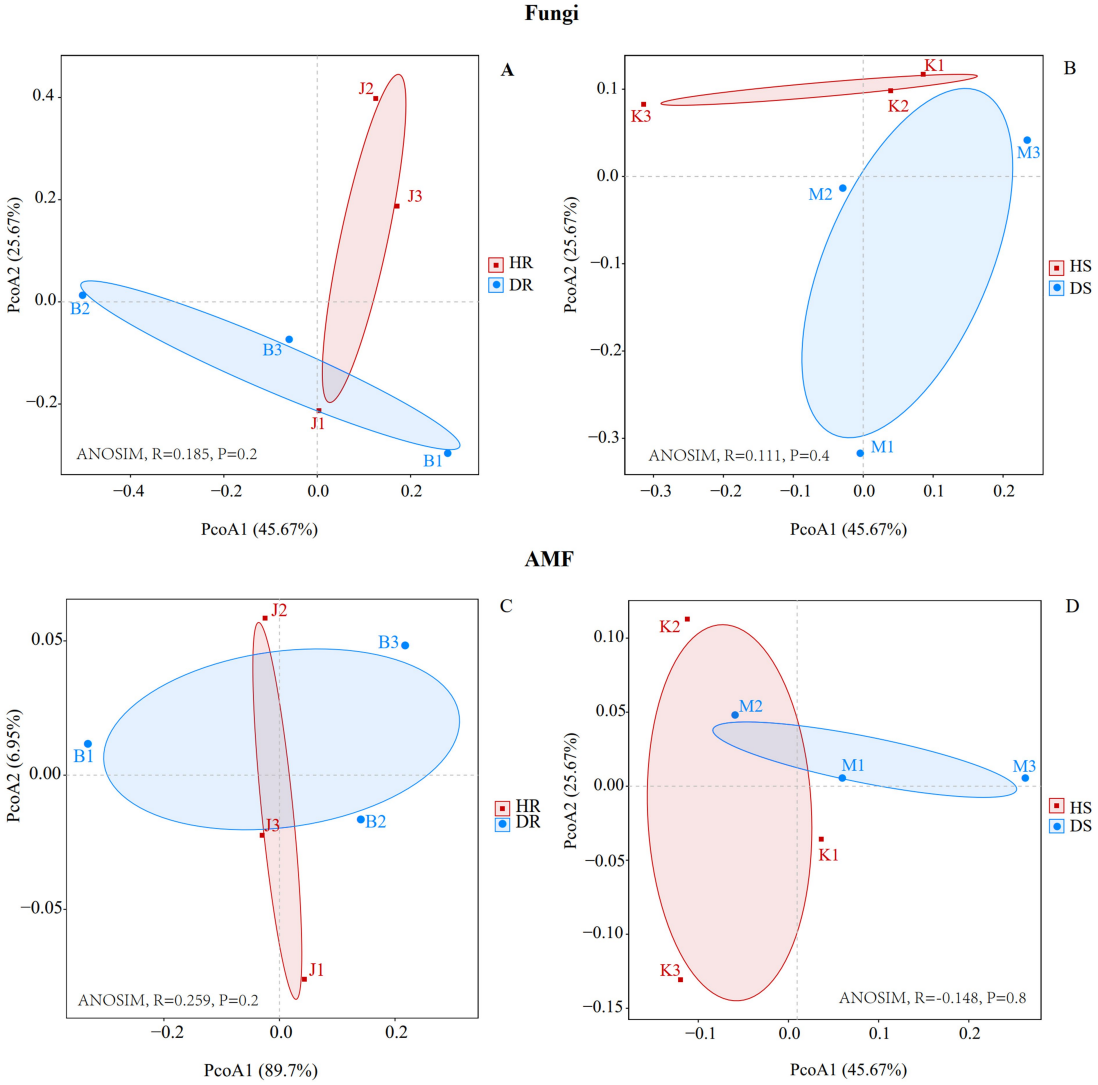
PCoA analysis of root **(A)**, rhizosphere soil **(B)** fungi communities and root **(C)**, rhizosphere soil **(D)** AMF communities at genus level. Healthy root (HR): J1/J2/J3. Diseased root (DR): B1/B2/B3. Healthy rhizosphere soil (HS): K1/K2/K3. Diseased rhizosphere soil (DS): M1/M2/M3.

From the Beta diversity principal coordinate analysis (PCoA) plot, the fungal community (Figures 1A,B) composition within each group—HR, DR, HS, and DS—was relatively consistent. The contribution rates of PCoA1 and PCoA2 were 45.67 and 25.67%, respectively. Regardless of whether the samples were from roots or rhizosphere soil, there was significant clustering differentiation between diseased and healthy samples. This indicates that root collar rot in pomegranate had a significant impact on the fungal communities in both roots and the surrounding rhizosphere soil. Principal coordinates analysis of AMF *β*-diversity revealed disease-induced community shifts in both roots and rhizosphere soil (Figures 1C,D).

#### 3.1.2 OTU clustering and fungal community composition analysis

A total of 1,472 fungal OTUs were identified from the diseased and healthy roots and rhizosphere soils. Based on a 97% sequence similarity threshold, fungal sequences were classified into 196–961 OTUs, primarily belonging to 4 phyla: Ascomycota, Basidiomycota, Mortierellomycota, and Chytridiomycota. These were further categorized into 10 classes, 23 orders, 36 families, and 41 genera.

**Figure 2:**
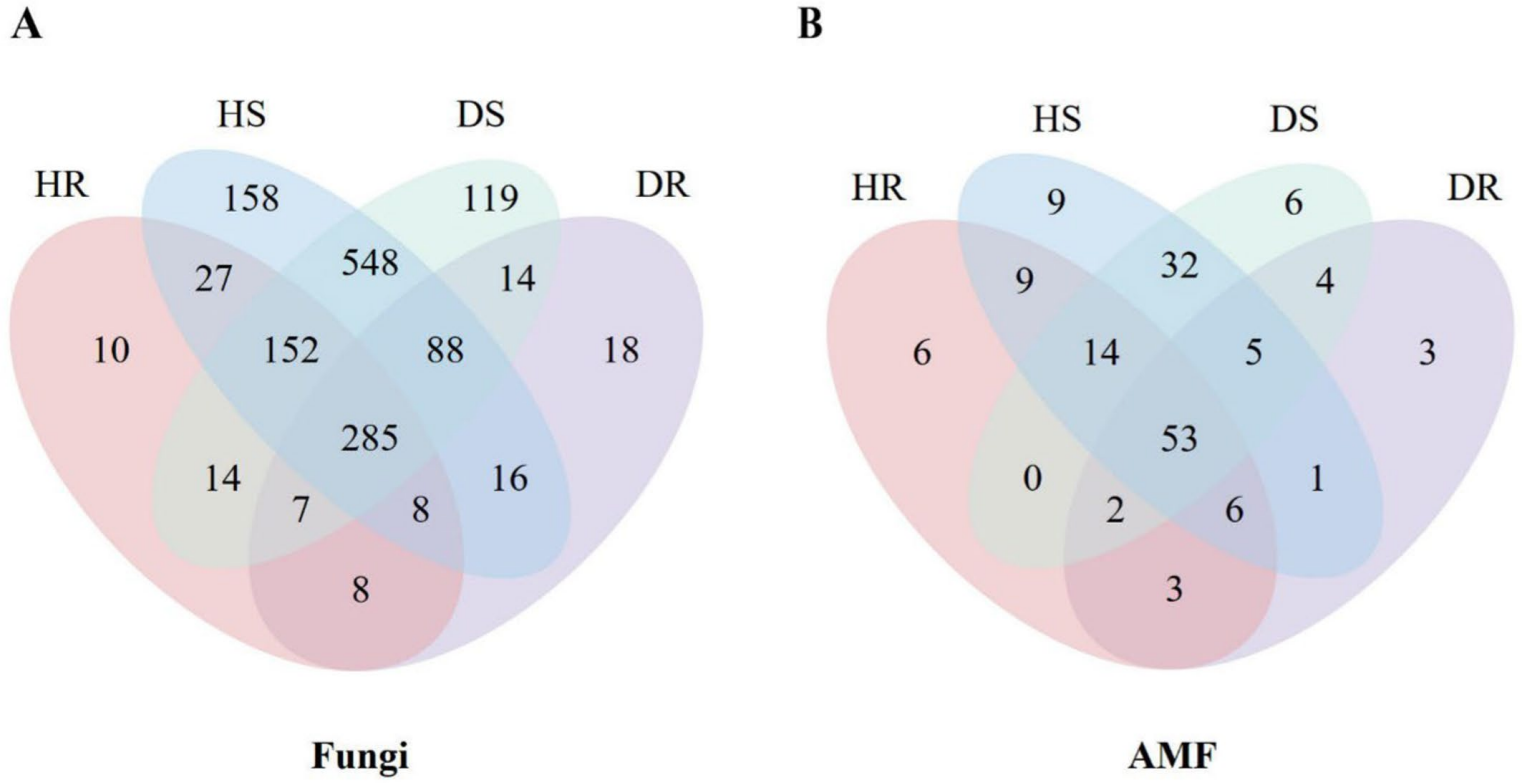
Venn diagram of fungi **(A)** and AMF **(B)** communities of root and rhizosphere soil at the OTU level.

A Venn diagram analysis (Figure 2A) of fungal OTUs in the four sample groups [healthy root (HR), diseased root (DR), healthy rhizosphere soil (HS), and diseased rhizosphere soil (DS)] revealed that 19.36% of OTUs were shared among all groups. The healthy root samples contained 10 unique fungal OTUs, annotated to genera such as *Pezicula* (a genus of inoperculate Discomycetes) and arbuscular mycorrhizal fungi including *Septoglomus*. In soil samples, a total of 1,436 OTUs were identified, with 14.55 and 8.29% of OTUs unique to healthy and rhizosphere soil, respectively, contributing to the overall richness. The dominant fungal taxa varied across different sample types. In healthy roots, the dominant genera were *Oliveonia* (15.44%) and *Dactylonectria* (12.25%), while in diseased roots, *Lauriomyces* (20.36%) and *Fusidium* (16.65%) were predominant. The diseased root samples contain 18 unique fungal OTUs, among which 5 genera of them annotated as *Amphobotrys*, *Paraconiothyrium*, *Phaeomoniella*, *Thyronectria* and *Truncatella* have been reported to cause plant diseases (Guarnaccia et al., 2022; Sokolova et al., 2022; Ji et al., 2023; Greeshma et al., 2024; Li et al., 2024). Venn analysis identified only one unique OTU of AMF (Figure 2B) present in diseased roots was assigned to *Paraglomus* (Supplementary Tables 1, 2).

### 3.2 Biomarkers for each sample

**Figure 3:**
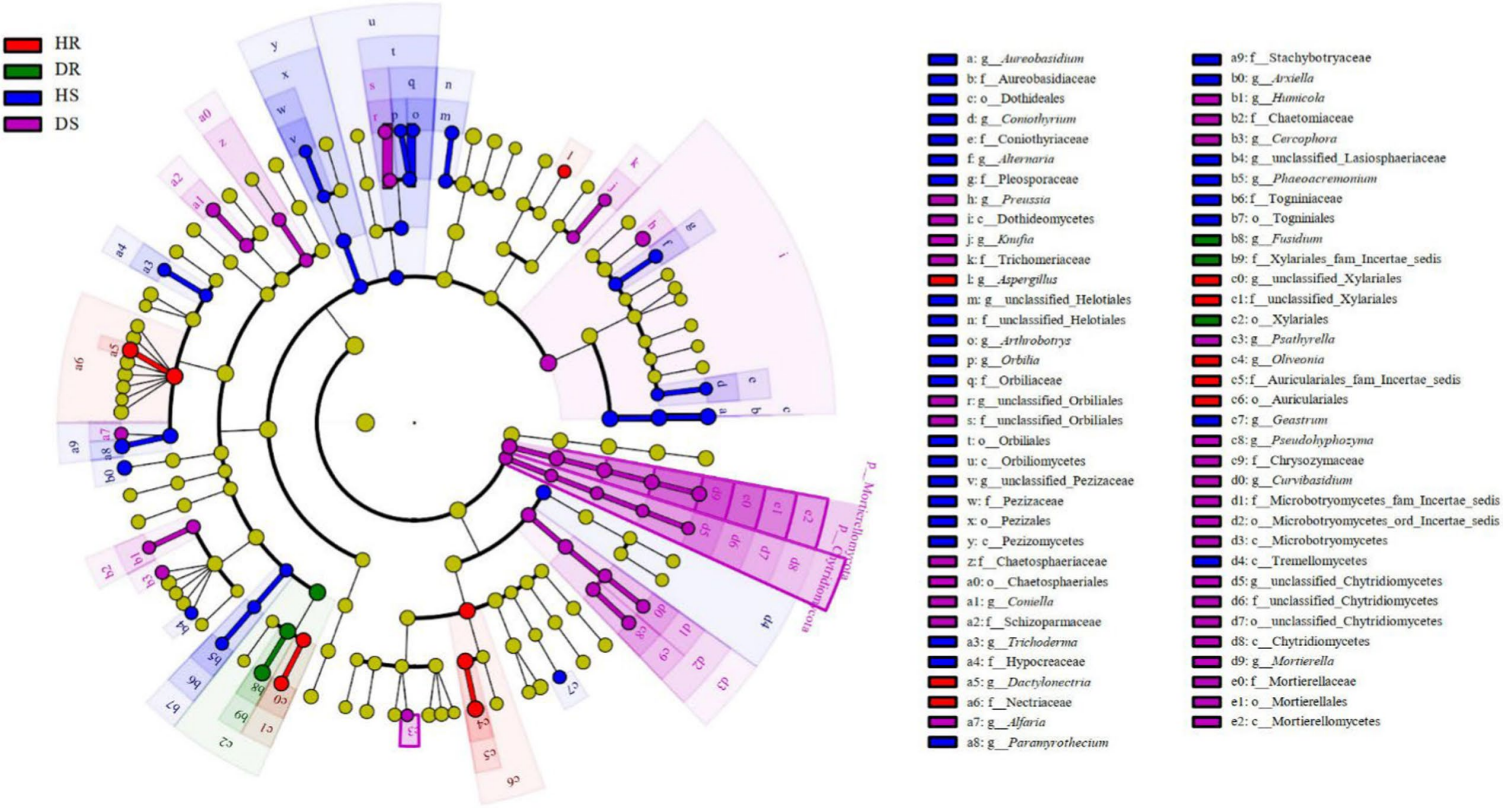
LEfSe analysis of root and rhizosphere soil fungi communities. The red, green, blue and purple nodes in the figure represented fungal taxa that were significantly enriched in HR, DR, HS and DS respectively, and significantly influenced the differences between groups at the same time. The yellow nodes represented nondifferentiated fungal taxa. The c, o, f and g represented class, order, family and genus, respectively.

During the transition of pomegranate from healthy to diseased states, fungal distribution in the roots and rhizosphere soils of healthy and diseased plants was compared. Distinct biomarker fungal taxa were identified in the rhizosphere soils of healthy (HS) and diseased (DS) plants (Figure 3). At the class level, Dothideomycetes, Microbotryomycetes, Chytridiomycetes, and Mortierellomycetes were significantly enriched in diseased rhizosphere soil (DS). At the order, family, and genus levels, the number of differential fungal taxa in rhizosphere soils was significantly higher than that in root samples. At the genus level, fungal taxa with significant intergroup abundance differences in healthy soil (HS) included: *Aureobasidium*, *Coniothyrium*, *Alternaria*, *Arthrobotrys*, *Orbilia*, *Trichoderma*, *Paramyrothecium*, *Arxiella*, *Phaeoacremonium*, and *Geastrum*. In diseased soil (DS), taxa with significant intergroup differences included: *Preussia*, *Epiphyte*, *Coniella*, *Alfaria*, *Humicola, Cercophora*, *Psathyrella*, *Pseudohyphozyma*, *Curvibasidium*, and *Mortierella*. In the roots, *Aspergillus*, *Dactylonectria*, and *Oliveonia* were significantly enriched in healthy roots, while *Fusidium* was significantly enriched in diseased roots. Among these taxa, *Aureobasidium*, *Alternaria*, *Paramyrothecium*, *Coniella*, *Dactylonectria*, and *Fusidium* were identified as potential plant pathogenic fungi. These taxa may play a critical role in the progression and severity of root collar rot in pomegranate.

**Figure 4:**
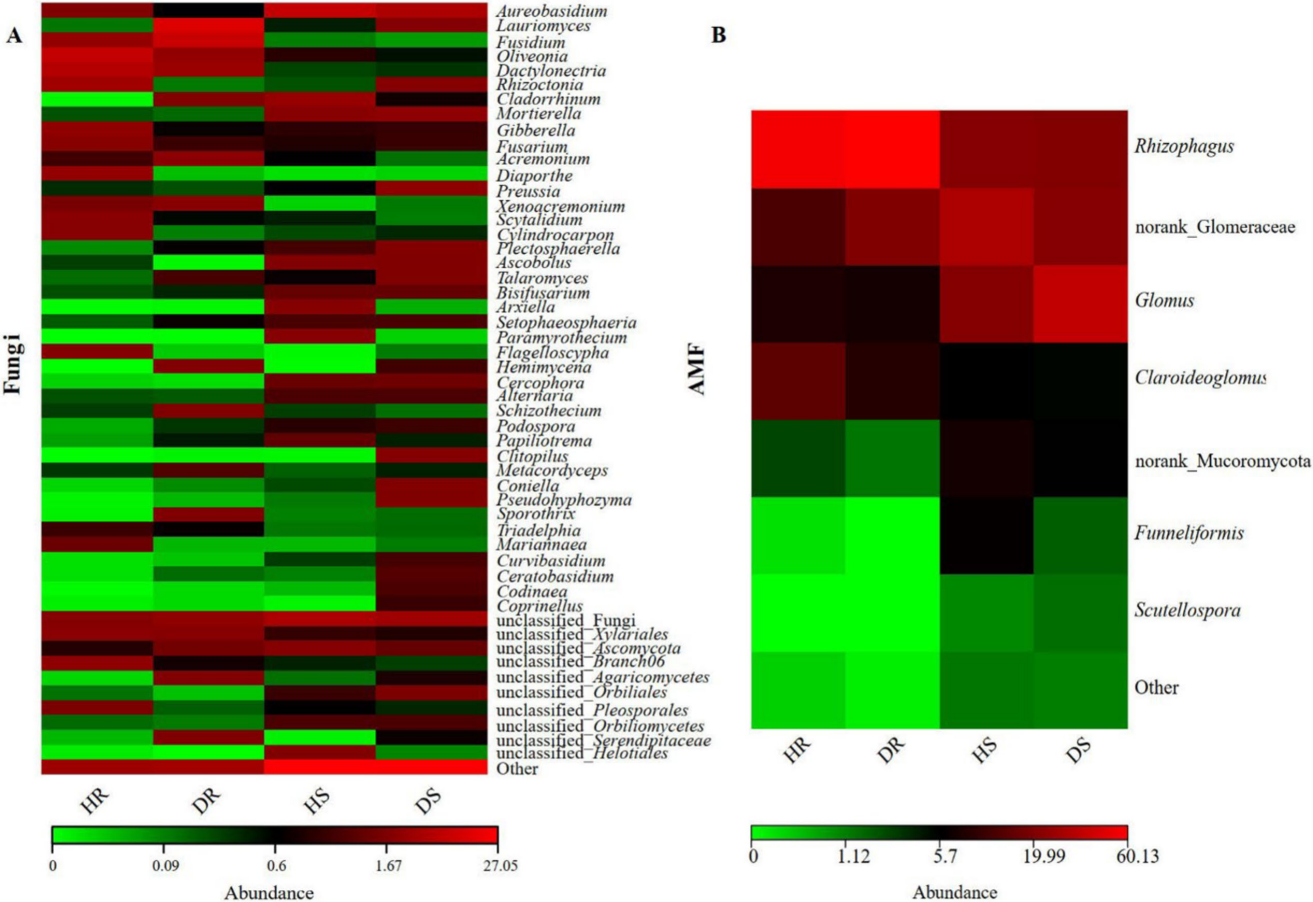
Heatmap of fungal **(A)** and AMF **(B)** abundance at the genus level for each sample.

In addition, a heatmap was created to study fungi with higher relative abundances at the genus level in each sample (Figure 4). In the diseased and healthy roots of pomegranate, there are also several other distinct fungal groups. For instance, pathogenic fungi such as *Rhizoctonia* sp., *Gibberella* sp., *Diaporthe* sp., *Plectosphaerella* sp., etc. (Morin et al., 2022; da Silva et al., 2024); biocontrol fungi including *Aureobasidium* sp., *Talaromyces* sp., *Metacordyceps* sp., etc.; and probiotic fungi like *Cladorrhinum* sp., *Papiliotrema* sp., etc. (Ali et al., 2022). These fungi play a role during the disease development process. Most AMF genera (beneficial symbionts) showed reduced abundance in diseased roots and rhizosphere soil. Interestingly, Glomus displayed contrasting trends: decreasing in roots but increasing in rhizosphere soil. This divergence warrants further investigation.

### 3.3 Functional prediction of fungal communities

**Figure 5:**
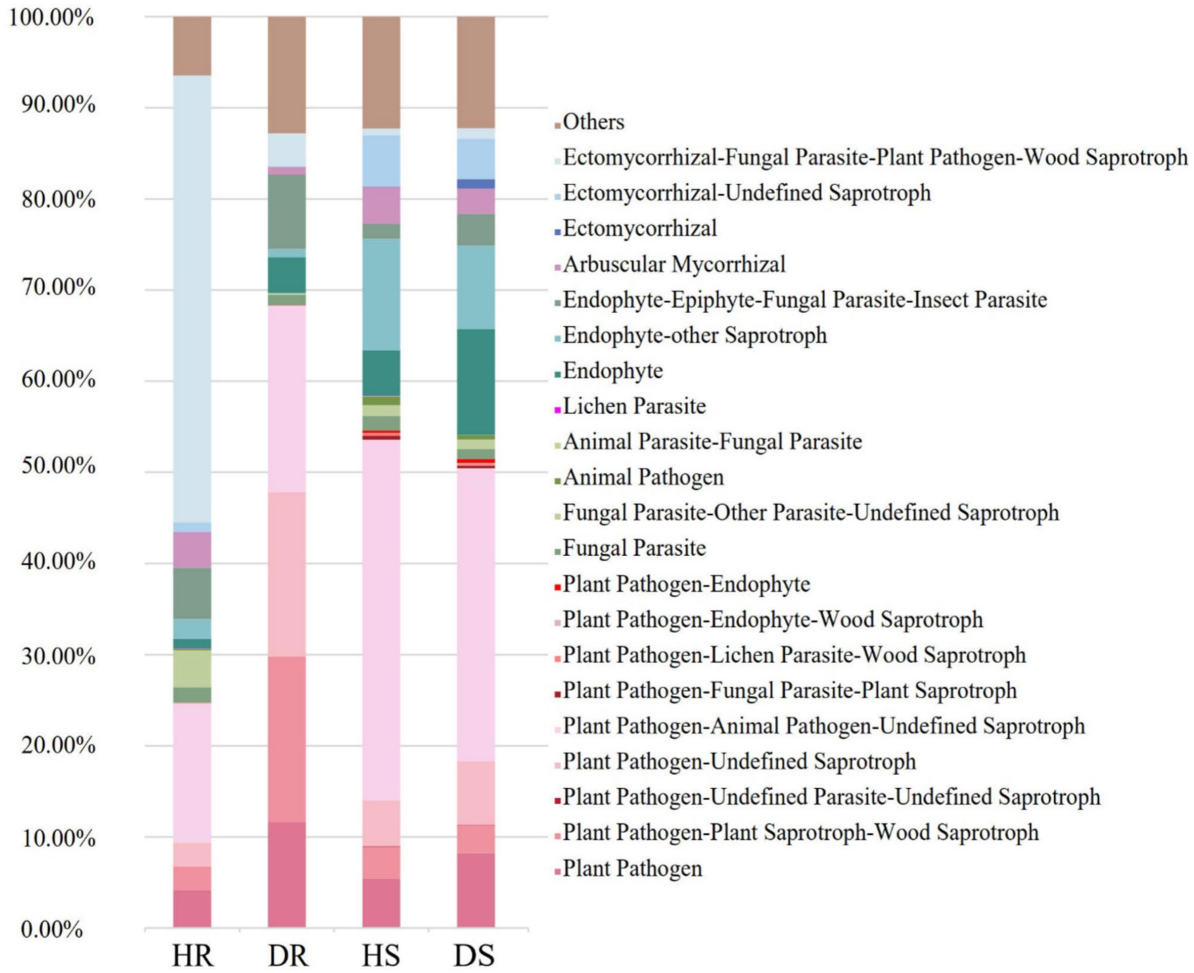
The nutritional structure of fungi in root and rhizosphere soil fungi communities. The ‘Others’ represented the sum of fungal taxa with relative abundance of less than 1%.

The functional roles of fungal communities in the pomegranate rhizosphere were predicted using FUNGuild 1.0 software. In healthy roots, the dominant functional group was ectomycorrhizal-undetermined saprotrophic fungi, with a relative abundance of 50.08%. After root collar rot infection, the relative abundance of this group decreased sharply to 3.69%. Similarly, the relative abundance of the arbuscular mycorrhizal functional group declined from 3.94% in healthy roots to 0.79% in diseased roots, with a similar decreasing trend observed in soil samples. Conversely, in diseased roots, the total relative abundance of plant-pathogen-related functional groups increased significantly to 68.36%, compared to 24.74% in healthy roots (Figure 5).

### 3.4 Influence of soil physicochemical properties on fungal community composition in the pomegranate rhizosphere

No significant differences were observed in the physicochemical properties of rhizosphere soil between healthy and diseased pomegranate. The rhizosphere soil had a neutral pH. Based on the soil nutrient grading standards from the Second National Soil Survey of China, all tested nutrient levels in the sampling site soil were categorized as Level 5 or Level 6, indicating high to extremely high nutrient availability (Table 2). These findings suggest that the sampling site’s soil is nutrient-rich and unaffected by root collar rot, potentially due to long-term artificial fertilization.

**Table 2:** Physicochemical properties of the rhizosphere soil of heathy and diseased pomegranate.

Sample Name	pH	Organic Matter (OM) (g/kg)	Available Phosphorus (AP) (mg/kg)	Available Potassium (AK) (mg/kg)	Total Nitrogen (TN) (g/kg)	Total Phosphorus (TP) (g/kg)	Total Potassium (TK) (g/kg)	${\mathrm{NO}}_{3}^{-}-N$ (mg/kg)	${NH}_{4}^{+}-N$ (mg/kg)
HS	7.80 ± 0.10	47.00 ± 5.35	116.53 ± 25.74	464.33 ± 68.71	1.93 ± 0.11	2.09 ± 0.06	19.60 ± 0.35	8.36 ± 1.11	40.64 ± 2.22
DS	7.73 ± 0.06	47.83 ± 1.76	121.47 ± 33.90	558.33 ± 88.08	2.04 ± 0.03	2.22 ± 0.29	18.87 ± 0.40	12.08 ± 0.66	52.30 ± 0.22

The data in the table were means ± SD (standard deviation). Different letters in the same column indicated significant differences among samples at *p* < 0.05 level.

**Figure 6:**
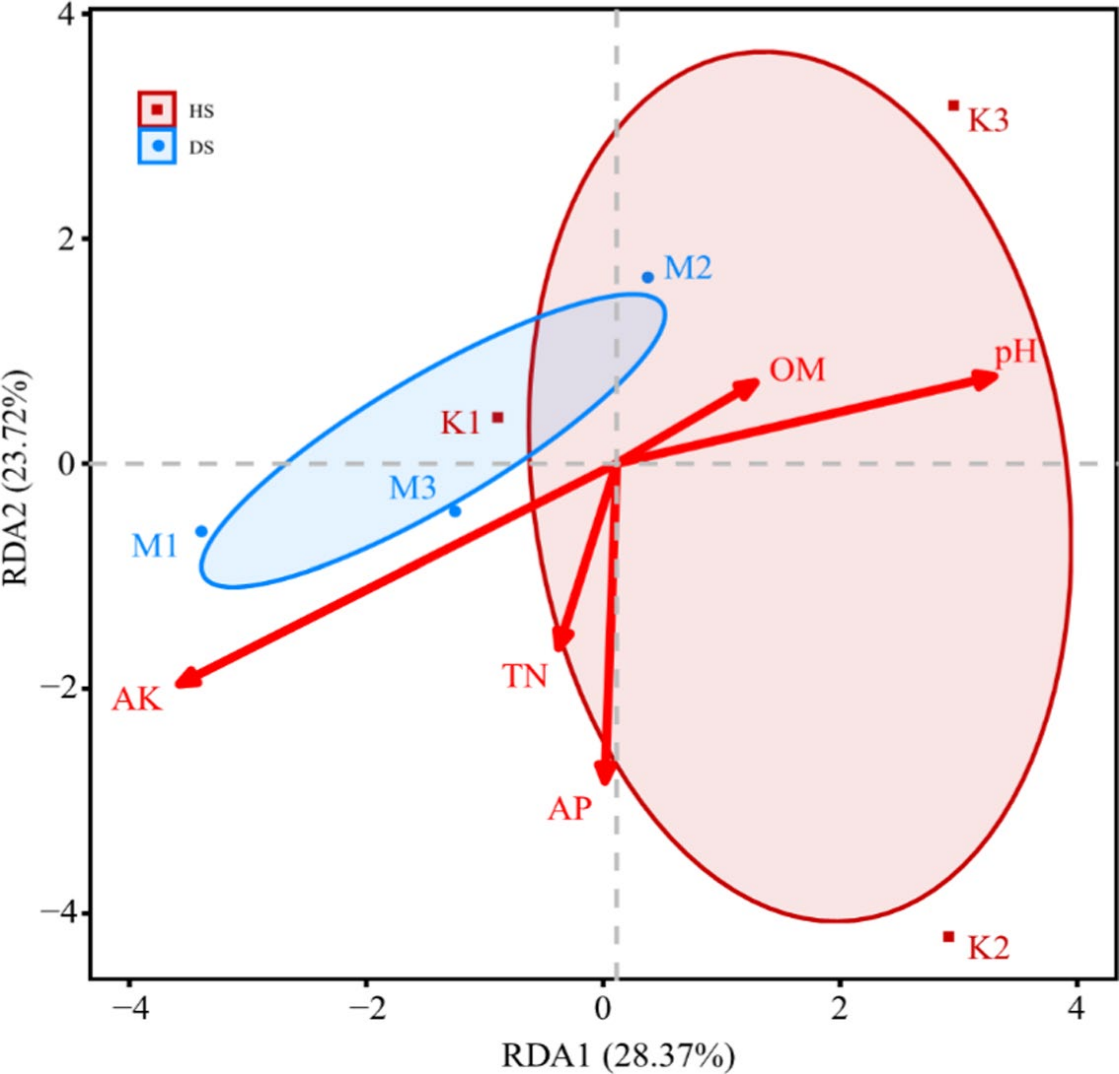
Effects of soil physicochemical properties on relative abundance at genus level. Healthy root (HR): J1/J2/J3. Diseased root (DR): B1/B2/B3. Healthy rhizosphere soil (HS): K1/K2/K3. Diseased rhizosphere soil (DS): M1/M2/M3.

Redundancy Analysis (RDA) of Soil Physicochemical Properties and Fungal Community Composition. Redundancy analysis was conducted by integrating data on the physicochemical properties of pomegranate rhizosphere soils and fungal communities. The cumulative contribution rates of RDA1 and RDA2 were 28.37 and 23.72%, respectively. Five soil physicochemical properties played significant roles in shaping the fungal community structure, with their influence ranked as follows: AK > pH > AP > TN > OM. Compared to healthy soils, diseased soil samples exhibited closer intra-group distances, indicating that the fungal community structure within diseased soils was more consistently influenced by soil physicochemical properties (Figure 6). The effects of AK and TN were more pronounced on the fungal community structure of diseased soils (e.g., M1, M3) compared to healthy soils (e.g., K1). In contrast, pH and OM primarily influenced fungal community composition in healthy soil samples.

**Figure 7:**
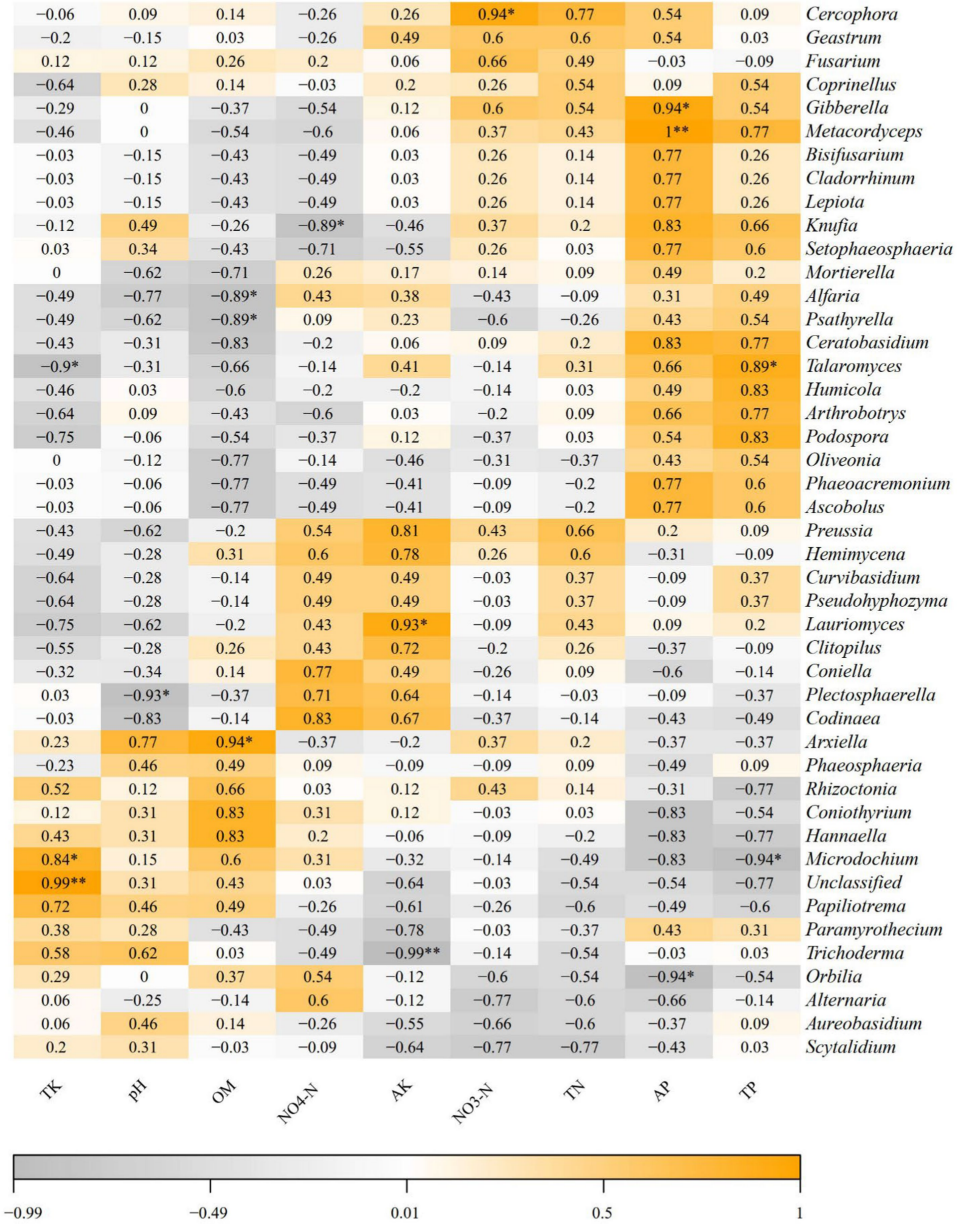
Spearman corrlation between rhizosphere soil fungi communities at genus level and soil physicochemical properties. The color represented the correlation. ^*^ and ^**^ indicate significant correlation at *p* < 0.05, *p* < 0.01, respectively.

Spearman correlation analysis based on soil physicochemical properties and Illumina MiSeq sequencing results was conducted to further investigate the relationships between soil physicochemical properties and dominant soil fungi at the genus level (relative abundance >1%) (Figure 7). The results indicated that pH was significantly negatively correlated with the genus *Plectosphaerella* (*p* < 0.05). Organic matter (OM) was significantly negatively correlated with *Alfaria* and *Psathyrella* (*p* < 0.05) but significantly positively correlated with *Arxiella* (*p* < 0.05). Ammonium nitrogen (NH₄^+^-N) was significantly negatively correlated with *Knufia* (*p* < 0.05). Available potassium (AK) was significantly positively correlated with *Lauriomyces* (*p* < 0.05) and highly significantly negatively correlated with *Trichoderma* (*p* < 0.01). Nitrate nitrogen (NO₃^−^-N) was significantly positively correlated with *Cercophora* (*p* < 0.05). Available phosphorus (AP) was significantly positively correlated with *Gibberella* (*p* < 0.05). These correlations directly reflect the structural composition of the fungal community.

### 3.5 Verification of pathogenicity and identification

#### 3.5.1 Pathogenicity tests

**Figure 8:**
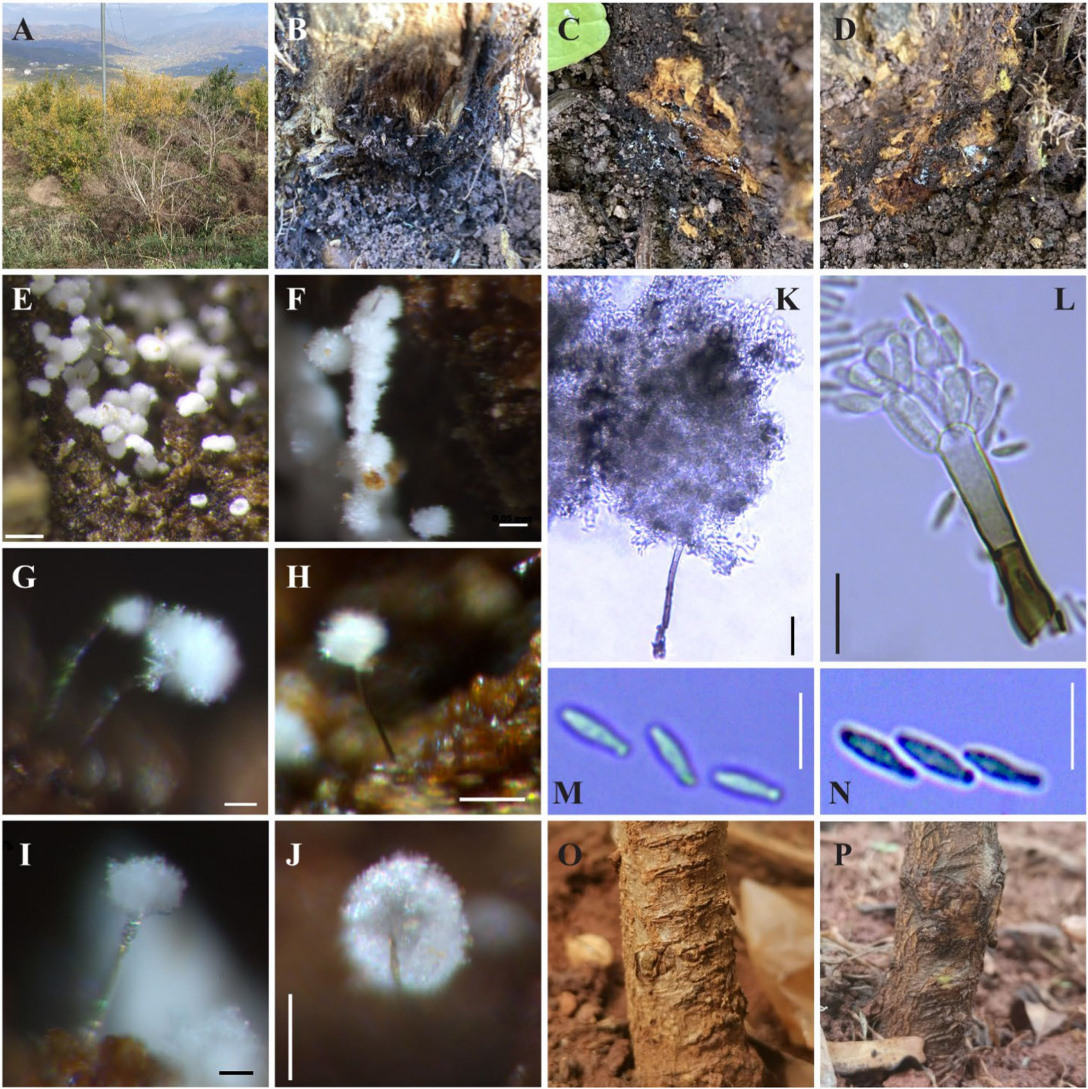
**(A)** Diseased pomegranate; **(B–D)** Symptom on pomegranate collar associated with *Lauriomyces bellulus*. **(E–J)** Conidiophore groups observed with stereomicroscope (Zeiss Discovery-V20, Leica M166FC); **(K,L)** Conidiophore groups observed with optical microscope (Leica DM2500); **(M,N)** Conidium; **(O,P)**: Inoculated seedings: **O**, control, **P**, symptoms caused by *L. bellulus*. Scale bars: **E** = 0.15 mm; **F**, **H**, **J** = 0.05 mm; **G**, **I** = 0.02 mm; **K** = 25 μm; **L–N** = 10 μm.

Approximately one-month post-inoculation, the inoculated seedlings exhibited necrotic lesions and decay symptoms. In contrast, the control seedlings remained symptom-free. To verify the identity of the pathogen, hyphae, and conidiophores were collected from the affected seedlings and subjected to reidentification through morphological and molecular biological techniques. These findings confirmed the presence of the initially inoculated pathogen, consistent with Koch’s postulates (Figures 8O–P).

#### 3.5.2 Identification of pathogen

The symptoms observed on the diseased collar of a pomegranate are depicted in Figures 8A–D. For morphological identification, *Lauriomyces bellulus* (P.W Crous & M. J. Wingfield anam., Figures 8E–N): hyphae were septate, branched, smooth and hyaline, becoming brown near the conidiophores. The conidiophore was macronematous, mononematous, simple, solitary, erect, smooth, septate, dark brownand thick-walled at the base, becoming thin-walled and light brown toward the apex, about 600 μm long, 4–7 μm wide. Ramoconidia blastic-acropetal, catenate, hyaline, smooth, cylindrical to ellipsoidal, rounding towards flattened, subtruncate ends, 6–9 × 1.5–3 μm, with up to 10 conidia in the main branches conidial showing thickened eonidial dehiscence scars (Crous and Wingfield, 1994).

**Figure 9:**
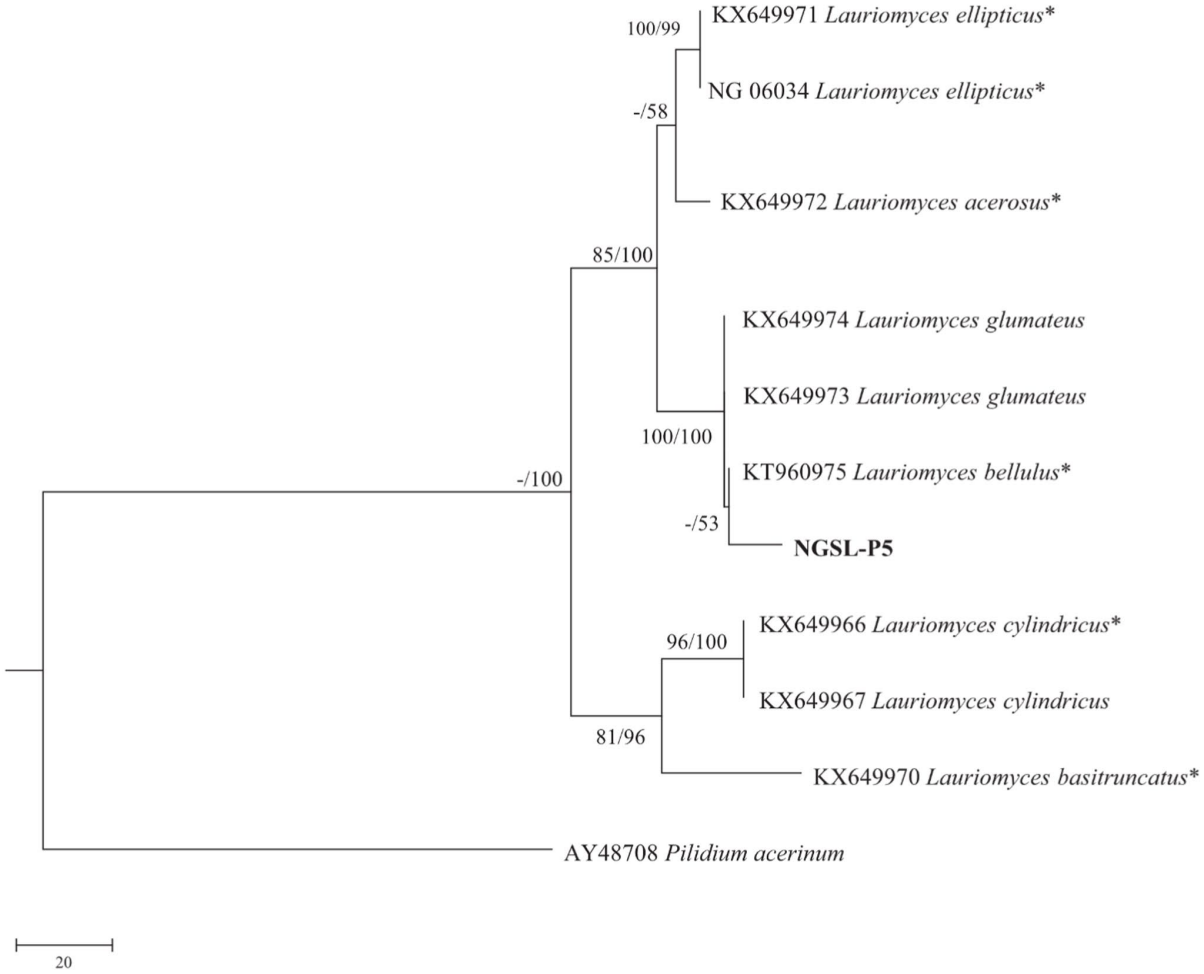
Maximum likelihood strict consensus tree illustrating the phylogeny of *Lauriomyces bellulus* and related species based on ITS, LSU and SSU sequences. Branches are labeled with maximum likelihood bootstrap higher than 70%, parsimony bootstrap proportions higher than 50%.

Molecular identification was performed using BLASTn searches against the ITS sequences. The sequence NGSL-P5 exhibited the highest similarity to *Lauriomyces bellulus*, with 99.39% identity (673/bp) compared to the type culture CBS 517.93 (GenBank Accession No. NR137151). The sequences have been deposited in GenBank with the following accession numbers: ITS: ON495704; LSU: ON495742; SSU: ON495748. Phylogenetic analyses utilizing the combined dataset (Table 3, Somrithipol et al., 2017) were conducted using Maximum Parsimony (MP) and Maximum Likelihood (ML) methods, as presented in Figure 9. Both morphological analysis and molecular characteristics confirmed the identification of NGSL-P5 as *Lauriomyces bellulus*.

**Table 3:** GenBank and culture collection codes related to *Lauriomyces* from this study.

Isolate	GenBank Accession LSU number			Culture code
	SSU	LSU	ITS1/2	
*Lauriomyces bellulus*	KT960974	KT960975	EF029218	ICMP 15050
Lauriomyces acerosus^*^	KX649961	KX649972	KX649983	CC00030
Lauriomyces basitruncatus^*^	KX649959	KX649970	KX649981	CC00049
Lauriomyces cylindricus^*^	KX649955	KX649966	KX649977	SFC:01649-1
*Lauriomyces cylindricus*	KX649956	KX649967	KX649978	SFC:01649–2
Lauriomyces ellipticus^*^	NG_065657	NG_060342	NR_155327	BCC 4007
Lauriomyces ellipticus^*^	KX649960	KX649971	KX649982	SFC:00424
*Lauriomyces glumateus*	KX649962	KX649973	KX649984	CC00037
*Lauriomyces glumateus*	KX649963	KX649974	KX649985	CC00064
*Pilidium acerinum*	AY487090	AY487089	AY487088	BPI 843554

^*^Holotype.

## 4 Discussion

### 4.1 Directional changes in microorganisms due to soil eutrophication

The dynamics of soil microbial core communities, including their growth, decline, and their capability to suppress root rot disease, are influenced by environmental factors. These factors also regulate the interactions between plant roots and soil microorganisms (Dasgupta and Brahmaprakash, 2021). In this study, the physicochemical properties of the soil associated with pomegranate did not show significant changes and exhibited eutrophic characteristics, which were associated with the anthropogenic application of chemical fertilizers.

Research has indicated that the long-term overapplication of chemical fertilizers severely threatens the health of soil ecosystems, alters soil nutrient dynamics, and consequently modifies microbial community structures, ultimately driving the soil ecosystem toward a pathological state (Wang et al., 2019). The accumulation of potassium (K), phosphorus (P), and nitrogen (particularly ammonium nitrogen, NH₄^+^-N, and nitrate nitrogen, NO₃^−^-N) can reduce microbial activity and diversity, disrupt nutrient absorption by microorganisms, and induce microbial cell apoptosis (Sahab et al., 2021; Shen et al., 2021; Yang et al., 2011). (1) The redundancy analysis results in this study revealed that the top five soil physicochemical properties influencing fungal community composition were, in descending order, available potassium (AK) > pH > available phosphorus (AP) > total nitrogen (TN) > organic matter (OM). In the rhizosphere soil of diseased plants, AK was the most influential soil physicochemical factor. The dominant genus Lauriomyces in the roots of diseased pomegranates showed a significant positive correlation with AK (*p* < 0.05). Pathogen invasion may cause root exudate changes that modulate rhizosphere microbiota, give rise to potassium-increasing, to trigger plant defense responses (Ansari et al., 2024; Chen et al., 2024). (2) AP was also a critical factor influencing fungal communities. A global study by Ma et al. (2023) found that the colonization rate and abundance of arbuscular mycorrhizal fungi (AMF) decreased significantly with increasing soil AP (*p* < 0.001). As an essential nutrient for plant growth, variations in AP can lead to changes in plant biomass, which may, in turn, alter fungal groups and nutritional conditions, causing competition among bacteria, soil animals, and fungi (He et al., 2016; Huang et al., 2016). Furthermore, AP can influence soil microorganisms by altering other soil physical and chemical properties, such as pH (Cai et al., 2018). (3) Compared to the healthy group, NH₄^+^-N content in diseased samples increased significantly (by 28.69%). NH₄^+^-N was significantly negatively correlated with the genus Knufia (*p* < 0.05). Knufia petricola is associated with ammonia oxidation processes and may influence soil physicochemical properties (Erdmann et al., 2022). High concentrations of NH₄^+^ can induce stress symptoms in some plants, including ionic imbalance, increased oxidative stress, and reduced biomass accumulation. Nitrogen forms in the soil can elicit differential responses between plants and microorganisms. For instance, under NO₃^−^ nutrient conditions, the endophytic fungus Phomopsis liquidambaris promotes Arabidopsis seedling growth, whereas under NH₄^+^ nutrient conditions, it inhibits seedling growth (Sun et al., 2022). (4) Field investigations revealed that orchards primarily rely on pumped tap water from mountain foothills for irrigation. Due to high elevation, water supply remains highly unstable. Combined with precipitation patterns in dry-hot valley climates, this leads to long-term alternation between drought and waterlogging in orchard soils. Concentrated rainfall or irrigation causes abrupt surges in soil moisture, potentially breaking pathogen dormancy and triggering disease outbreaks. Moreover, irrigation water may carry pathogens for field dispersal, exacerbating disease spread (Zhang and Li, 2021).

In summary, the long-term application of chemical fertilizers has led to soil eutrophication, preventing plants from adjusting to the rhizosphere fungal community structure to meet their growth requirements. This disruption ultimately destroys fungal community homeostasis, resulting in the degradation or even deterioration of microbial communities (Lu et al., 2022; Lei et al., 2024). This may be one of the primary reasons for the severe occurrence of pomegranate collar rot disease in the study region. This study represents the first systematic analysis of microbial community structure differences in pomegranate root collar rot within dry-hot valley regions. It should be noted that this study does not cover regions with different climatic conditions or soil types, which may limit the universality of the conclusions. Given that this pomegranate variety is primarily cultivated in dry-hot valley areas of Yunnan Province, China, we maintain that these limitations do not diminish the reference value of current conclusions for similar ecological zones. However, special attention should be given to necessary adaptive modifications during practical applications based on regional characteristics.

### 4.2 Synergistic effects of *Lauriomyces bellulus* with rhizosphere fungi in accelerating pomegranate root collar rot

The structural integrity and diversity of soil microorganisms are pivotal in maintaining soil health and quality. Pathogenic microorganisms, capable of surviving in soil for extended periods, can propagate through soil particles, roots, and water. This propagation may initiate new diseases, trigger regional epidemics, and cause chain reactions that heighten the risk of crop diseases. Rhizosphere microorganisms, in particular, are significant reservoirs of soil-borne pathogens, which include those responsible for seedling damping off (Philippot et al., 2013; Zhalnina et al., 2018).

ITS-based fungal identification has limitations and cannot rely solely on Illumina MiSeq sequencing to determine the pathogen of a certain disease. Therefore, this study still adopted the Koch’s postulates to verify the pathogen of pomegranate rot collar rot. The combination of Illumina MiSeq sequencing results helped narrow down the range of fungi to be tested. For complex and difficult to identify plant diseases, amplicon sequencing can effectively identify potential pathogenic regions by analyzing the differences between microbial communities in diseased and healthy plants, thereby saving time in formulating pathogen-specific control measures (Mukuma et al., 2020; He et al., 2022). In this study, less than 20% of microbial taxa were shared between healthy and diseased pomegranate root samples, with significant changes in dominant genera. The healthy roots were significantly enriched with genera such as *Aspergillus* and *Oliveonia*, while the relative abundance of *Lauriomyces* and *Fusidium* (the sexual stage of which belongs to *Nectria*) increased significantly in diseased roots (*p* < 0.01).

Among these genera, *Aspergillus* and *Oliveonia* are primarily saprotrophic. *Lauriomyces sakaeratensis* has been identified as a pathogen causing fruit rot in Dipterocarpus (Somrithipol et al., 2006). Some fungi in the genus *Nectria* are common plant pathogens, capable of causing a variety of stem and root diseases (Keith et al., 2010; Li, 2017; Račko et al., 2020). The pathogen responsible for pomegranate root collar rot was confirmed to be *Lauriomyces bellulus* through pathogenicity tests, demonstrating that amplicon sequencing can significantly expedite the identification of diseases, especially for pathogens that are challenging to cultivate on artificial media. In addition, this is the first report of collar rot of pomegranate caused by *L. bellulus* in China.

In healthy roots, various pathogenic fungi maintain a dynamic equilibrium. However, when *Lauriomyces bellulus* dominates a specific ecological niche and produces Haplofungins—compounds with potent inhibitory effects on fungal inositol phosphoceramide synthase (IPC)—it suppresses other pathogenic fungi. This leads to an increased abundance of single colonies, which in turn causes plant disease (Ohnuki et al., 2009). Furthermore, there was a notable increase in the abundance of the *Plectosphaerella* in the roots of diseased plants. This genus includes several fungi known to cause root diseases, particularly *P. melonis*, which is associated with root rot in various crops. *Lauriomyces bellulus*, may in conjunction with *Plectosphaerella* sp., plays a role in accelerating the development of pomegranate root collar rot. But this inference still needs to be verified through later experiments.

### 4.3 Rhizosphere microorganisms as a vital reservoir of beneficial microbes

Rhizosphere microorganisms represent a crucial reservoir of beneficial microbes. Probiotic organisms in the rhizosphere can synergistically extract nutrients from the soil, bolster plant growth, and fortify plant resistance against pathogens (Das et al., 2021; Huang et al., 2025). Shifts in microbial community structures are known to directly influence the prevalence and severity of soil-borne diseases (Jayaraman et al., 2021; Chauhan et al., 2023). In response to threats such as *Lauriomyces bellulus* infection, pomegranate roots can recruit antagonistic fungal communities that not only combat this pathogen but also enhance overall tree vigor by fostering the aggregation of probiotic communities.

Although constrained by data acquisition dimensions such as sample time series, this study has not yet established direct correlation models with continuous succession processes of soil health status. Nevertheless, it reveals that pomegranate plants exhibit reduced abundance of beneficial biological communities after pathogen infection, while showing a trend of recruiting beneficial microorganisms through pathogen interaction. This aligns with the theory proposed by van der Heijden et al. (2016). FUNGuild analysis revealed that the healthy rhizosphere fungal community was predominantly composed of ectomycorrhizal-undefined saprotrophic functional groups and arbuscular mycorrhizal (AM) functional groups. Mycorrhizal fungi not only benefit plant growth and development but also enhance host resistance, improve soil fertility, and maintain soil health (Liu and Chen, 2007). Specific genera in healthy roots included *Pezicula* and AM fungi such as *Glomus* and *Septoglomus*. Secondary metabolites of *Pezicula* fungi, such as Pezicumin, exhibit inhibitory activity against nine plant pathogens, including *Botrytis cinerea* and *Passalora fulva* (Wang et al., 2014). *Aureobasidium* is a genus with a relatively high abundance in healthy roots, where *A*. *Pullulans* is well-recognized as a mature biocontrol agent. However, a notable decline in the abundance of this genus has been observed in samples from diseased roots. This reduction could impact on the natural biocontrol mechanisms within the rhizosphere, potentially diminishing the soil’s disease-suppressive capabilities.

Following the onset of collar rot disease, the relative abundance of ectomycorrhizal-undefined saprotrophic and AM functional groups declined, while the abundance of pathology-related functional groups increased. Despite the decline in certain beneficial fungi like *Aureobasidium*, there are still other beneficial fungal groups that have undergone a degree of enrichment. Research indicates that in the early stages of plant disease, changes in root exudates may recruit or enrich beneficial microorganisms as a form of self-rescue (Bakker et al., 2018; Lyu and Smith, 2022; Feng et al., 2024). These fungi play various roles, including biocontrol, promoting plant growth, and improving soil conditions, which collectively contribute to the plant’s ability to combat disease and stress. Dominant genera in diseased root samples were mostly saprotrophic Basidiomycota fungi, including *Pluteus*, *Lepiota*, *Tremella*, and *Thyronectria*. Saprotrophic fungi contribute to organic matter degradation, facilitating carbon and nitrogen cycling in soil, which can enhance or improve soil organic matter and nutrient composition (Clocchiatti et al., 2020). Additionally, unique fungal taxa in diseased pomegranate roots included *Fusidium* and *Mortierella*. Secondary metabolites of *Fusidium* fungi, such as fusidic acid, act as antibiotics against certain fungal pathogens (Huang et al., 2021). *Mortierella* fungi contribute to soil nutrient improvement through phosphate solubilization (Osorio and Habte, 2013) and the degradation of herbicides such as diuron (Ellegaard-Jensen et al., 2013) and endosulfan (Kataoka et al., 2010; Li et al., 2018). In the diseased root samples, there is an observed increase in the abundance of non-endemic beneficial fungi, encompassing five genera. Notably, the relative abundance of *Talaromyces* has increased by 1.05%. The ready-to-use dry-powder formulation of *T. flavus* Bodhi001 demonstrates an effective inhibitory effect against rice brown leaf spot disease, highlighting its potential as a biocontrol agent (López-Moral et al., 2021; Volynchikova and Kim, 2022; Jantasorn et al., 2025). Other fungi such as *Acremonium alternatum* and *Metacordyceps chlamydosporia* have been effectively reported to prevent and control plant diseases in both greenhouse and field conditions (Auer et al., 2024). Additionally, *Aureobasidium pullulans* has been developed into a relatively mature biocontrol agent (Cignola et al., 2024). Some fungi compete with plant pathogens for livable space and nutrients, such as *Papiliotrema flavescens*, which exerts antifungal effects through potential competition for nutrition, space, and parasitism and parasitism. Another species, *P*. *huenov*, improves soil conditions by decomposing heavy metals (Nguyen et al., 2020; Nguyen Van et al., 2021). In pot experiments, *P. laurentii* showed significant effects on Brinjal growth promotion and biocontrol of *Fusarium* wilt, indicating a beneficial role in plant health management (Das et al., 2023). Fungal communities play critical roles in disease suppression not only in green plants but also in mushroom crops. For example, Yu et al. (2025) indicated that inoculating the *Morchella importuna* mycosphere with *Pseudomonas chlororaphis* increased the *α*-diversity of the soil fungal community and suppressed the abundance of the pathogen *Paecilomyces penicillatus*, leading to reduced white mould disease incidence and increased morel yield. Thus, there is a discernible relationship between the differential changes in these fungal groups and pomegranate root and neck rot disease, and that manipulation of beneficial microorganisms could be a broadly applicable strategy in disease management.

The colonization of microbial strains has long been considered a major limiting factor affecting the effectiveness of biocontrol strains. Extensive research suggests that microbial communities possess resilience and resistance, capable of quickly reverting to their original structure following external disturbances or the introduction of new species (Beisner et al., 2003; Toju et al., 2018; Hayashi et al., 2024). Consequently, changing established microbial community types is often challenging, especially when a single or a few strains are introduced into an existing microbial system without continuous supplementation, leading to their rapid replacement by other existing microorganisms (Castro-Sowinski et al., 2007; Luo et al., 2019; Quiroz, 2022). Reports suggest plants can “perceive and respond to their environment” through genetic experience, emitting specific chemical signals to attract mobile organisms that meet their needs, thereby achieving self-protection (Spoel and Dong, 2012; Leopold, 2014; Das et al., 2021). The enrichment behavior of beneficial fungi during the “healthy-diseased plant” transition in pomegranates may relate to this mechanism. During this process, organic matter released by rhizosphere microbes becomes incorporated into soil organic matter, and the soil develops memory of these dynamic changes (Lapsansky et al., 2016). Rhizosphere microbes from plants successfully adapted to environments can persist and influence subsequent plants even after original plant removal. If more comprehensive experiments could validate this hypothesis, selected microbes might be used to construct beneficial microbial communities within the “ecological memory of plants and rhizosphere soil fungi,” potentially circumventing challenges caused by microbial communities’ inherent resilience and resistance. Therefore, utilizing Illumina MiSeq sequencing to compare the differences in rhizosphere microorganisms between diseased and healthy plants, and based on ecological memory of plants and rhizosphere soil fungi, and selecting core microbial communities that are beneficial for plant growth and disease resistance from the inherent microbial community of the environment, constructing a rhizosphere microbial community assembly (MCA), and transforming soil from a susceptible state to a disease inhibiting state, can be an effective strategy for preventing this disease (Tian et al., 2024).

## 5 Conclusion

This research investigated the roots and rhizosphere soils of healthy and collar rot-affected pomegranate, analyzing the relationship between collar rot disease and the diversity and structural composition of rhizosphere fungal communities. The rhizosphere of healthy plants exhibited high species richness, with no dominant potential pathogenic fungi, indicating a relatively stable community structure. However, after infection, the distribution of rhizosphere fungal communities became more homogenized, the balance of the rhizosphere fungal community structure was disrupted, and soil nutrient composition shifted, transitioning from a probiotic to a pathological state.

The prevention and control of soil-borne diseases present significant challenges. Traditional chemical pesticide methods offer limited efficacy against soil pathogens and can contribute to environmental pollution. As a result, integrated management strategies are typically employed to address soil-borne diseases. The findings clarify the impact of collar rot disease on the structure of pomegranate rhizosphere fungal communities, providing a basis for further research into the disease’s pathogenic mechanisms. Meanwhile, this work also offers theoretical support for identifying biocontrol microorganisms to manage pomegranate collar rot.

## Data Availability

All raw data for this study is available on the NCBI database (accession number: PRJNA1170523): https://www.ncbi.nlm.nih.gov/search/all/?term=PRJNA1170523.

## Supplementary material

SUPPLEMENTARY Table 1 Otu_core_spec.groups.

Supplementary Table 2 Otu_taxonomy.
